# The efficacy of virtual distance training of intensive therapy and anaesthesiology among fifth-year medical students during the COVID-19 pandemic: a cross-sectional study

**DOI:** 10.1186/s12909-021-02826-1

**Published:** 2021-07-22

**Authors:** Enikő Kovács, András Kállai, Gábor Fritúz, Zsolt Iványi, Vivien Mikó, Luca Valkó, Balázs Hauser, János Gál

**Affiliations:** Department of Anaesthesiology and Intensive Therapy, Semmelweis University, P.O.B. 2, H-1428, Budapest, Hungary

**Keywords:** COVID-19, Virtual learning, Medical education, Intensive therapy, Anaesthesiology

## Abstract

**Background:**

The coronavirus disease (COVID-19) brought several challenges in medical education. The aim of our study was to investigate whether virtual distance trainings (VDT) organized during the COVID-19 pandemic at our university were effective in replacing in-person bed-side education in intensive therapy and anaesthesiology among fifth-year medical students, both from students’ and instructors’ perspectives.

**Methods:**

This was a cross-sectional study consisting of three parts: a 20-item students’ questionnaire filled out by students participating in VDT, a 22-item instructors’ questionnaire filled out by instructors taking part in virtual distance education and a 20-item knowledge test completed by students participating in VDT, as well as by students visiting bed-side trainings (BT) during the same semester, before COVID-19 pandemic. The questionnaires focused on effectiveness, content, self-preparedness, technical background and interactivity of VDT. Instructors’ and students’ responses given to the common questions, as well as the knowledge test results were compared. Mann-Whitney U test was used for group comparisons and binary logistic regression was performed to analyze the influence of previous health-care experience on students’ feeling of self-preparedness.

**Results:**

One hundred thirthen students (response rate {RR}: 68%) and 29 instructors (RR: 97%) filled out the questionnaires. The majority of students found our VDT useful and effective; however, a considerable number of participants felt disadvantaged by taking VDT instead of BT sessions and would recommend keeping virtual distance education methods combined with BT. Instructors found VDT overall effective and deemed the transfer of their knowledge satisfactory; however, they described worse interactivity and contact with students during virtual sessions compared to in-person teaching. Instructors showed a clearer consensus that VDT should not replace BT in the future, while students’ answers were more divided in this regard. Previous health-care experience did not influence students’ feeling of self-preparedness.

One hundred and twenty-seven students (56 after VDT {RR: 34%}; 71 after BT {RR: 67%}) completed the end-of-semester knowledge test. Students attending VDT performed better than students visiting BT (median score VDT:83.5 vs BT:77.3; *p* = 0.015).

**Conclusions:**

Virtual distance learning incorporating virtual practice sessions was effective in maintaining continuous education of intensive therapy and anaesthesiology among fifth-year medical students during the COVID-19 outbreak.

**Supplementary Information:**

The online version contains supplementary material available at 10.1186/s12909-021-02826-1.

## Background

The COVID-19 (coronavirus disease 2019) pandemic brought several challenges not only for health care, but for economics, education and everyday life in general. Several countries, including Hungary, chose to introduce drastic measures to slow down the spread of the virus and to “flatten the curve” based on the recommendations of the World Health Organization (WHO) [1]. One of these actions was the temporary closure of universities with the option of either creating a rapidly developed online educational material and/or postponing in-person education until the improvement of the epidemic situation [2].

Intensive therapy and anaesthesiology (ITA) education faces several difficulties during the COVID-19 outbreak. Countries affected by a large number of COVID-19 cases suffer from lack of human resources in the intensive care units (ICU), diminishing teaching capacity. In addition, this is a field requiring not only theoretical knowledge, but clinical skills and hands-on experience as well, hence the transition of the curriculum to a fully e-learning platform is quite difficult even at the undergraduate level [3, 4].

Hungary declared a state emergency due to the COVID-19 pandemic on March 11, 2020. The national regulations restricted all levels of in-person education at Hungarian universities from March 11 to May 25, 2020. In an effort to manage this unexpected emergency situation and continue the intensive therapy and anaesthesiology education among fifth-year medical students, we modified the presentation of our curriculum and introduced online virtual distance trainings (VDT) instead of bed-side trainings (BT). In addition, face-to-face lectures were transformed to online virtual lectures. However, the efficacy of this rapidly introduced educational method was uncertain.

The aim of our study was to investigate whether VDTs as a part of our intensive therapy and anaesthesiology virtual distance education were able to replace in-person bed-side education methods among fifth-year medical students during the COVID-19 pandemic. Additionally, we also analyzed if virtual sessions could be incorporated into our course in the future based on our students’ and instructors’ impressions and opinions.

## Methods

### Study design and participants

We performed a cross-sectional study (STROBE checklist - Additional file 1) to evaluate the effectiveness of virtual practical sessions in the compulsory ITA course among fifth-year medical students both in the Hungarian and German language program at Semmelweis University Budapest, Hungary. The study consisted of three parts. An anonymous and voluntary internet-based survey was conducted among our students participating in thematic VDTs and virtual distance education during the COVID-19 outbreak. Moreover, instructors in charge of virtual sessions during the COVID-19 pandemic were also interviewed through an online questionnaire. The instructors’ questionnaire was also anonymous and voluntary. Additionally, a multiple-choice knowledge test was filled out on a voluntary basis by students who took part in the virtual practices and also by students, who completed the corresponding practical sessions in the same semester during our traditional bed-side education prior to the restrictions implemented because of the COVID-19 pandemic. The test results of the two student groups were compared.

The Semmelweis University Regional and Institutional Committee of Science and Research Ethics approved our study (Approval nr.: 101/2020). Questionnaires and tests were completed anonymously. Potential participants were informed that by completing the questionnaire and/or test they would provide consent for anonymized data analysis and potential publication.

### The intensive therapy and anaesthesiology (ITA) course

The traditional ITA compulsory course contained five 90-min long thematic BTs and four 90-min long simulation trainings (Additional file 2: Table 1) within a one-week block schedule for each group, supplemented with fourteen 70-min long lecture sessions held weekly during the spring semester of the 2019/2020 academic year. Lectures were presented in the form of face-to-face education until the introduction of COVID-19 restrictions and were switched to the virtual form of education during the COVID-19 restrictions.

As Additional file 2: Table 1 shows, students visited intensive care unit during “Introduction”, “Respiratory”, “Shock” and “Trauma” thematic BTs. They examined patients and were introduced to the equipment related to the topic of a particular practice. “Anaesthesiology” BT was held in an operating room, where students could observe the operation of an anaesthesia machine, and the equipment used during anaesthesia.

The student to instructor ratio was 7:1 or less both during BTs and simulation trainings.

The final exam was administered as an oral exam during the examination period between May 18 and July 3, 2020.

### Virtual distance education during the COVID-19 outbreak

Virtual distance education in the form of VDTs and lectures was introduced as a necessity into our curriculum in the middle of the spring semester of the 2019/2020 academic year. The remaining lectures and four of our thematic BTs (“Introduction”, “Respiratory”, “Shock” and “Anaesthesia” trainings) were adapted to virtual sessions. The “Trauma” session was cancelled due to its specific patient population and lack of human resources during the pandemic. Simulation trainings and one BT in the ICU were postponed and then held after the withdrawal of restrictions at the end of the semester, in the same manner as before the restrictions.

Virtual lectures took place through Zoom® (San Jose, California, USA) platform in the same time points, utilizing the same structure as previous face-to-face lectures. All students participating in the ITA course this semester, regardless of the type of trainings they visited, had an opportunity to take part in six face-to-face and eight virtual lectures.

The four thematic VDTs were conducted at specific time points for a given group through the internet using Zoom® platform. A maximum of 21 students took part in one virtual 90-min long training session. A brief PowerPoint® (Microsoft, Redmond, USA) presentation summarizing the principles of the trainings’ topic, videos and images showing patients and patient management composed the main framework of a virtual practice with the opportunity of further discussion with students. Furthermore, case reports were incorporated into the VDTs to enhance the practical aspect of a session. The same topics were covered as during traditional BTs (Additional file 2: Table 1). Student engagement was enhanced by using the Poll Everywhere® (San Francisco, USA) application. Moreover, online articles and videos were uploaded to Moodle® (Perth, Australia) learning management system to support learning and understanding of a given topic.

Final exams were conducted in the same manner for students who completed the traditional and virtual distance learning courses.

**Fig. 1:**
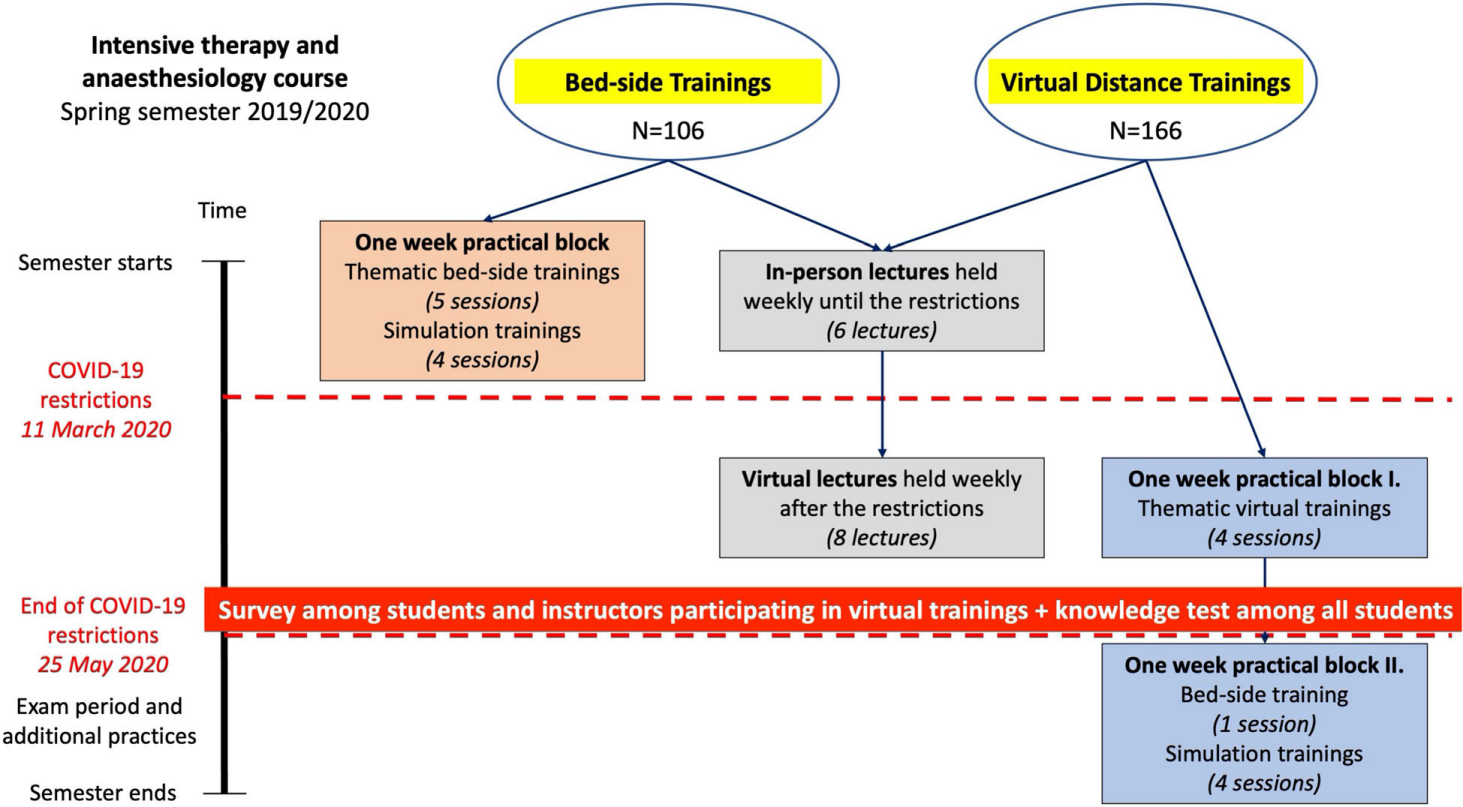
The structure of intensive therapy and anaesthesiology course before and after COVID-19 restrictions. The intensive therapy and anaesthesiology course consisted of a scheduled one-week practical block with five thematic bed-side trainings and four simulation trainings, supplemented with fourteen lectures held weekly during the spring semester of the academic year 2019/2020. Lectures were presented in the form of face-to-face education until the introduction of COVID-19 restrictions and were switched to virtual form of education during the COVID-19 restrictions. Bed-side trainings were suspended with the introduction of COVID-19 restrictions and were replaced with virtual distance trainings. Students who had the scheduled one-week practical block during the COVID-19 restrictions, took part in four thematic virtual trainings that replaced thematic bed-side trainings. They attended the lectures in the same way as students visiting bed-side trainings. In order to assess the success of the virtual distance training with the four thematic sessions, a survey was filled out by students and instructors participating in virtual distance trainings on a voluntary and anonymous basis. In addition, all students visiting our course during the spring semester of 2019/2020 had the opportunity to complete a knowledge test to compare the knowledge of bed-side training and virtual distance training students. The participation in the knowledge test was also voluntary and anonymous. The simulation trainings and one bed-side training were held for students visiting virtual distance trainings after the lifting of restrictions

The structural differences of ITA course before and after the COVID-restrictions can be seen in Fig. 1.

### Students’ and instructors’ questionnaire

We developed a 20-item questionnaire with 14 Likert-scale questions (graded from 1 to 5, where 1 indicated a strong disagreement and 5 a strong agreement), two open-ended questions and four multiple-choice questions (Additional file 2: Table 2) regarding the four thematic VDTs for our students. The Likert-scale questions and open-ended questions covered the quality of the four VDTs, students’ self-report of their preparedness to recognize critically ill patients or being able to manage perioperative situations after completing the virtual sessions, as well as students’ opinion about changing bed-side learning to virtual education in the future. Multiple choice questions asked about the students’ demographics (age; gender; previous experience in health system).

The instructors received a 22-item questionnaire regarding virtual distance education, listed in Additional file 2: Table 3. 14 Likert-scale questions (graded from 1 to 5, where 1 indicated a strong disagreement and 5 a strong agreement) and two open-ended questions covered the quality, strengths and weaknesses of virtual sessions, as well as instructors’ opinion about introducing virtual education into our course in the future. Six multiple choice questions asked about demographics (age; gender; experience in education; experience in e-learning as educators; language program; job description).

The questionnaires went through an internal and external validation process based on the recommendations by the Association for Medical Education [5]. First, three senior educators reviewed both students’ and instructors’ questionnaires regarding content and intelligibility after the questionnaire development. As a next step, six educators taking part in VDTs evaluated the students’ questionnaire, which was followed by a written cognitive interview with ten Hungarian and five German students. The instructors’ questionnaire was assessed by six external educators experienced in virtual distance education, followed by a written cognitive interview with six instructors of our institute. Validation steps and timing of the questionnaires are specified in Additional file 2: Table 4.

### Knowledge test

A 20-item internet-based multiple-choice test was developed to assess students’ knowledge about the topics covered by the four thematic trainings (“Introduction”, “Respiratory”, “Shock” and “Anaesthesia” sessions). The questions were reviewed by three experts and educators in the field of anaesthesiology and intensive therapy. Each question consisted of five possible answers and maximum 100 points could be reached. Time for the knowledge test was maximized in 30 min. Both students participating in the traditional BTs and students taking part in VDTs received the test at the end of the semester, when all students completed the thematic trainings, before the oral exams. Taking the test was voluntary and the results did not influence final grades.

### Statistical analysis

The results of the questionnaires were analyzed using descriptive statistics. We determined the medians and interquartile ranges of the grades at each Likert-scale question, which were graded from 1 to 5. Additionally, continuous variables (age; knowledge test scores; time to complete knowledge test) were described as medians and interquartile ranges (IQR). Categorical variables (gender; language program; students’ previous job experience in health care; students’ previous work with critically ill patients; instructors’ experience in education and e-learning; instructors’ job description) were described as numbers and percentages.

The open-ended responses went through a content-analysis and were summarized into categories.

Less than 5% of data were missing; the missing data were excluded from the analysis.

Mann-Whitney U test was applied to compare the answers to four Likert-scale questions between students and instructors regarding the effectiveness and future application of VDTs.

In addition, Mann-Whitney U test was used to compare the knowledge test results of students who participated in the traditional bed-side practices and the ones who underwent virtual practice sessions.

Moreover, a binary logistic regression analysis was performed to determine if previous job experience with critically ill patients has an influence on students’ self-reported preparedness after completing the course (Additional file 3: Table 1).

The level of significance was set at *p* < 0.05.

Statistical analysis was performed using SPSS v25.0 (SPSS Inc., Chicago, IL). Figures were created by GraphPad Prism version 8.3.0. (GraphPad Software, La Jolla, CA).

## Results

Table 1 shows the number of students participating in our compulsory ITA course in the spring semester of the 2019/2020 academic year, the number of students and instructors taking part in VDTs and the response rate of the corresponding questionnaires and knowledge test.

**Table 1:** Number of students and instructors participating in the ITA course in the spring semester of the 2019/2020 academic year

	Total	VDT	BT
	N (%)	N (%)	N (%)
Students, spring semester 2019/2020	272 (100%)	166 (100%)	106 (100%)
Hungarian language program	161 (59%)	108 (65%)	53 (50%)
German language program	111 (41%)	58 (35%)	53 (50%)
**Questionnaire RR**		**113 (68%)**	
**Knowledge test RR**		**56 (34%)**	**71 (67%)**
Instructors, spring semester 2019/2020^a^	43 (100%)	30 (100%)	20 (100%)
Hungarian language program	31 (72%)	23 (77%)	20 (100%)
German language program	29 (67%)	15 (50%)	12 (60%)
**Questionnaire RR**		**29 (97%)**	

*VDT* Virtual distance training, *BT* Bed-side training, *N* Number of students, *RR* Response rate; questionnaire and knowledge test response rates are marked in bold

^a^ The majority of instructors participated both in VDT and BT, as well as in the Hungarian and German language program

Demographic data of responders are presented in Tables 2 and 3.

**Table 2:** Demographic data of students completing the questionnaire

Students	Median (IQ range) or N (%)
Total	113 (100%)
Age	24 (23,25)
Gender	
Female	78 (69%)
Male	35 (31%)
Previous job experience (ICU, ED or EMS)	
Yes	10 (9%)
Limited	16 (14%)
No	87 (77%)
Previous job experience with patient contact	
Yes	18 (16%)
Limited	33 (29%)
No	62 (65%)
Language program	
Hungarian	74 (66%)
German	39 (34%)

IQ Interquartile range, *N* Number of students, *ICU* Intensive care unit, *ED* Emergency department, *EMS* Emergency Medical System

**Table 3:** Demographic data of instructors completing the questionnaire

Instructors	Median (IQ range) or N (%)
Total	29 (100%)
Age	35 (30,45)
Gender	
Female	17 (59%)
Male	12 (41%)
Educator experience	
0–5 years	5 (17%)
5–10 years	11 (38%)
> 10 years	13 (45%)
Experience in E-learning as educator	
Yes - regular	2 (7%)
Yes - rare	12 (41%)
No	15 (52%)
Language program	
Hungarian	14 (48%)
German	6 (21%)
Both	9 (31%)
Job description	
Resident	8 (28%)
Consultant	13 (45%)
Academic position	8 (28%)

*IQ* Interquartile range, *N* Number of students

### Students’ questionnaire

As can be seen in Table 4, the students found our thematic VDTs useful and effective with an acceptable structural and technical quality. However, the majority of participants felt disadvantaged by taking the VDTs as opposed to the traditional BT and would not recommend replacing BT with VDT in the future. Nonetheless, they suggested to keep virtual distance education in combination with bed-side practical trainings.

**Table 4:** Students’ and instructors’ answers given to Likert-scale questions

Likert-scale questions	Students’ answers ***N*** = 113	Instructors’ answers ***N*** = 29	***P***
	Median (IQR)	Median (IQR)	
I received from the virtual distance training exactly what I have expected	4 (4, 5)		
The virtual practices perfectly replaced the bed-side sessions - there is no need for the latter.	2 (1, 3)	1 (1, 1)	*< 0.001*
The virtual practices are useful but strictly in combination with bed-side sessions.	5 (3, 5)	5 (5, 5)	*0.036*
I can recognize a patient with respiratory insufficiency after completing the virtual training.	4 (3, 4)	N/A	
I can recognize a patient with shock after completing the virtual training.	4 (3, 5)	N/A	
I am aware of the perioperative management after completing the virtual training.	4 (3, 5)	N/A	
Students were disadvantaged by taking the virtual practices instead of the bed-side sessions.	3 (3, 4)	4 (4, 5)	*0.012*
To what extent have the virtual practices raised your interest in intensive therapy and anaesthesiology?	4 (4, 5)	N/A	
To what extent was it easy to follow the flow of the virtual practices?	5 (4, 5)	N/A	
The virtual training was overall effective.	4 (3, 5)	4 (2, 4)	0.162
To what extent have you found the virtual practices’ imaging acceptable?	5 (4, 5)	N/A	
To what extent have you found the virtual practices’ audio acceptable?	5 (4, 5)	N/A	
To what extent have you found the material on Moodle® platform helpful?	4 (4, 5)	N/A	
To what extent have you found the virtual trainings acceptable in total?	5 (4, 5)	N/A	
I received sufficient information regarding distance learning from my institution.	N/A	4 (4, 5)	
I received sufficient materials regarding the virtual course from my institution.	N/A	4 (4, 5)	
Plenty of technical problems arose during virtual education leading to quality decrease of our teaching.	N/A	2 (1, 2)	
I received sufficient technical support during virtual education from my institution.	N/A	5 (4, 5)	
I could achieve a good interaction with students during virtual education – the same as during in-person sessions.	N/A	2 (1, 2)	
Students’ interactivity was satisfactory during virtual sessions – it reached the level of an in-person session.	N/A	2 (1, 4)	
I could transfer the planned knowledge during virtual teaching.	N/A	4 (3, 4.5)	
I enjoyed virtual distance learning despite the difficulties of the new situation caused by the COVID-19 outbreak.	N/A	4 (2, 5)	
The virtual trainings should be built into our course in the future.	N/A	4 (4, 5)	
I would never teach at virtual distance learning again.	N/A	1 (1, 1)	

Answer options to statements: 1.0: strongly disagree, 2.0: disagree, 3.0: undecided, 4.0: agree, 5.0: strongly agree. Answer options to questions: 1.0: definitely not, 2.0: probably not, 3.0: undecided, 4.0: probably, 5.0: definitely. The answers to common questions were compared between students and instructors. Mann-Whitney U test was used for comparison. Significant p-values are indicated in italics. *IQR* interquartile range, *N/A* non-applicable, as this group did not receive the particular question

Most of the responders reported a positive feeling of self-preparedness in recognizing respiratory failure, shock and in being able to manage perioperative situations after completing the VDTs. Previous experience with critically ill patients did not influence the students’ feeling of self-preparedness (Additional file 3: Fig. 1).

**Fig. 2:**
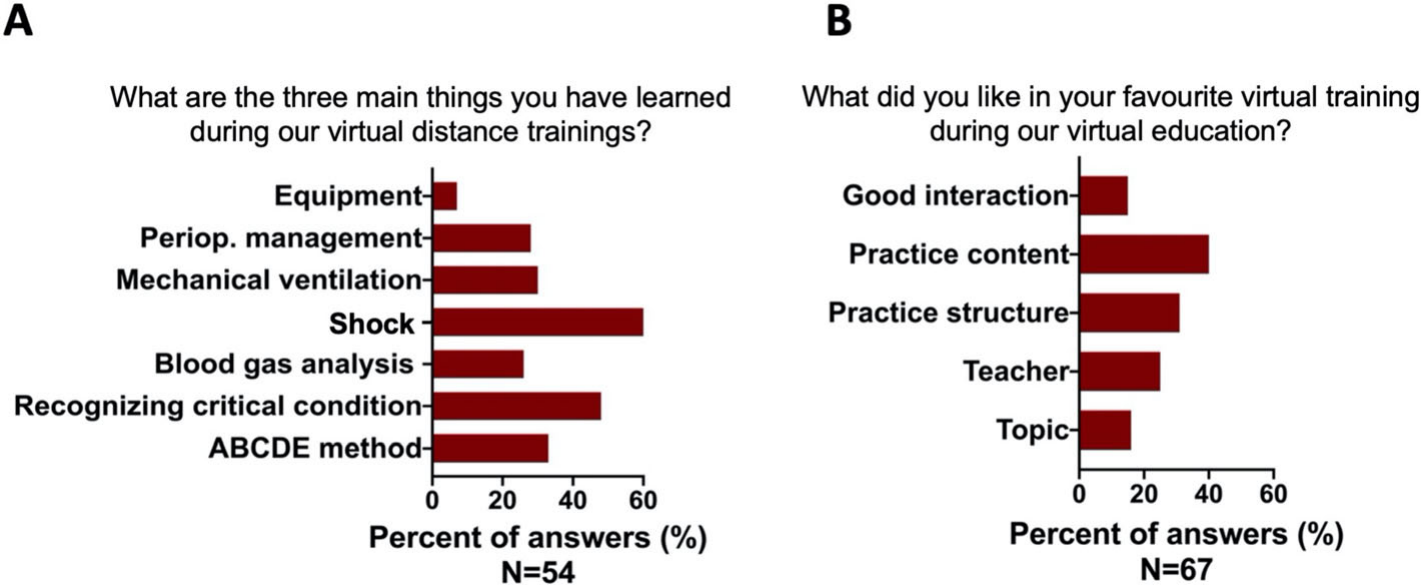
**A** and **B** Students’ answers given to open-ended questions. **A**: “What are the three main things you have learned during our virtual distance trainings?” was answered by 54 students. **B**: “What did you like in your favourite virtual training during our virtual education?” was answered by 67 students

The open-ended questions were answered only by a small group of responders (67 {40%} and 54 {32%} answers, for the two questions respectively). However, satisfactory content, structure and interactivity were achieved during the VDTs based on the answers (Fig. 2A and B).

### Instructors’ questionnaire

The medians and IQR of instructors’ answers to Likert-scale questions are shown in Table 4. As per the responses, the instructors received sufficient information and technical support to teach online. They found VDTs overall effective and reported a satisfactory transfer of their knowledge. However, they described worse interactivity and engagement with students during virtual distance education compared to in-person teaching.

**Fig. 3:**
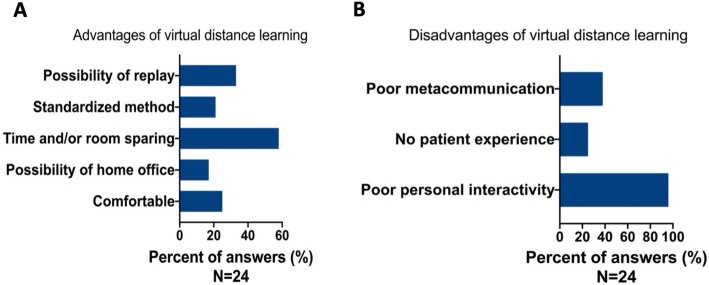
**A** and **B** Instructors’ answers given to open-ended questions. **A** and **B**: 24 educators (80% response rate) answered this part of questionnaire

The instructors’ answers to open-ended questions are shown in Fig. 3A and B.

As can be seen in Table 4, instructors were significantly more likely to conclude that students were disadvantaged due to VDTs compared to traditional bed-side learning, than students (instructors’ median answer [MA] {IQR}: 4 {4,5}; students’ MA {IQR}: 3 {3,4}; *p* = 0.012). However, as Table 4 shows, both groups found the VDTs effective to the same extent (instructors’ MA {IQR}: 4 {2,4}; students’ MA {IQR}: 4 {3,5}; *p* = 0.162). Although this difference is not significant, there is some shift in IQR, indicating that students tended to be more satisfied with the VDT. Additionally, instructors showed a clear consensus that VDTs should not replace in-person education in the future, while students’ answers were more divided in this regard (Table 4). Instructors were significantly more likely to conclude that VDTs did not replace bed-side sessions (instructors’ MA {IQR}: 1 {1,1}; students’ MA {IQR}: 2 {1,3}; *p* < 0.001), as well as VDTs are useful in a combination with BTs (instructors’ MA {IQR}: 5 {5,5}; students’ MA {IQR}: 5 {3,5}; *p* = 0.036).

### Knowledge test

The knowledge test was completed by 34% of students participating in VDTs and by 67% of students taking part in BTs (Table 1). The demographic data of students participating in knowledge test and their test results are shown in Table 5. Students completing the VDTs scored significantly higher during the test than students taking part in in-person BTs (VDT median score {IQR}: 83.5 {74.2, 88.7}; BT median score {IQR}: 77.3 {69.4, 83.6}; *p* = 0.015).

**Table 5:** Demographic data and test results of students participating in knowledge test

	Total	VDT	BT	p
	Median (IQ range)	Median (IQ range)	Median (IQ range)	
	or	or	or	
	N (%)	N (%)	N (%)	
Total	127 (100%)	56 (100%)	71 (100%)	
Gender				0.400
Female	80 (63%)	33 (59%)	47 (66%)	
Male	47 (37%)	23 (41%)	24 (34%)	
Language program				0.339
Hungarian	78 (61%)	37 (66%)	41 (58%)	
German	49 (39%)	19 (34%)	30 (42%)	
Test score	80 (71.3, 85.8)	83.5 (74.2,88.7)	77.3 (69.4, 83.6)	*0.015*
Test duration (min)	13.5 (10, 17)	13 (10, 17)	14.5 (10, 17)	0.292

*BT* Bed-side training, *IQ* Interquartile range, *N* Number of students, *min* Minutes, *VDT* Virtual distance training; significant *p* values are indicated in italics

## Discussion

The objective of our study was to examine the effectiveness of virtual distance learning and VDTs held instead of in-person bed-side ITA trainings for fifth-year medical students at our university during the COVID-19 pandemic. Furthermore, we explored the students’ and instructors’ opinions about introducing at least some elements of virtual distance education into our course in the future.

The restrictions implemented to control the COVID-19 outbreak resulted in several challenges in education and particularly in health-care education [6]. A number of countries simply suspended education at their universities, others redeveloped their courses to continue education on online platforms, or allowed face-to-face teaching with some special measures [7–11]. Furthermore, a significant impact of the COVID-19 pandemic was shown in regards to medical student education, particularly in the transition from student to doctor [12].

In-person education was suspended from the middle of March 2020 until the end of May 2020 in Hungary, with the option of virtual distance education or postponing courses. Our institute changed the presentation of ITA curriculum and chose to develop a hybrid course with VDTs and virtual distance lectures during the restrictions combined with in-person simulation sessions and one visit at the ICU delayed until restrictions were lifted. Intensive therapy and anaesthesiology is a medical field requiring a complex curriculum even in undergraduate education covering cognitive and technical skills, as well as teaching valuable communication skills and overall attitude in a specialized environment [13]. Conveying these skills using only virtual distance education is challenging; however, previous data has shown that teaching clinical skills through online education is as effective as traditional in-person learning [14], although experiencing the atmosphere of an intensive care unit is also crucial in the education of medical students [15].

Students participating in in-person practices met critically ill patients with the previously mentioned conditions (Additional file 2: Table 1). The biggest challenge during the transition of BTs to VDTs was to maintain “hands-on patient experience” and the practical aspect of our previous bed-side sessions. To enhance the efficacy and pragmatism of VDTs, case reports and discussions with videos and/or photos were used. The benefit of videoconferencing and e-learning systems in medical education has previously been shown, with the opportunity of not only delivering lectures or tutorials, but to present invasive procedures as well [16, 17]. In addition, an online focus group qualitative study found a good acceptance rate of synchronized online learning by students during the COVID-19 pandemic [18].

Both students and instructors found our VDTs effective and acceptable in replacing in-person learning and bed-side sessions to keep education continuous during the COVID-19 pandemic. However, neither group of participants supported the withdrawal of BTs in intensive therapy and anaesthesiology in the future showing the demand for hands-on patient contact and the in-person experience of the ICU atmosphere. Both students and instructors reported that students not participating bed-side practices during the COVID-19 outbreak were disadvantaged compared to students taking part in bed-side teaching; however, this view was significantly more pronounced for that of the instructors’. Moreover, instructors were significantly more likely to conclude that students were disadvantaged due to VDTs compared to traditional BTs than students. The possible cause of these differences is multifactorial. On the one hand, fifth-year medical students were younger than instructors in our study, representing a generation who is more familiar with online platforms and more experienced in using communication technologies offered by computers and the internet. On the other hand, instructors’ previous experience with in-person and hands-on education situations might have emphasized the lack of metacommunication and proper interactivity between students and instructors during virtual distance education, which are important elements of successful teaching [19]. We need to highlight that instructors had no complaints about technical problems during virtual sessions and they were overall satisfied with this type of education.

Students reported a satisfactory feeling of preparedness in recognizing respiratory failure or shock and managing patients in a perioperative setting after completing the VDTs. Nonetheless, we need to express that these reports are subjective and do not contain objective measurements of the real preparedness of students.

Furthermore, as an interesting finding, students participating in VDTs instead of in-person BTs performed significantly better at the end-of-semester multiple-choice test than students taking part in bed-side teaching. This result supports the fact that our VDT was successful in replacing in-person education during the COVID-19 outbreak; however, multiple-choice tests measure theoretical knowledge instead of practical skills and attitude, which might have been transferred less efficiently. Answers given to open-ended questions by our students indicate that the quality of teaching remained acceptable with adequate structures and methods during trainings. In our experience, VDTs permitted a more structured, uniform way of teaching, potentially leading to a more precise transmission of theoretical knowledge.

### Limitations

Our study has some limitations which need to be considered. First of all, our study is monocentric and only students and instructors of our institution are represented. The number of participants is small; however, questionnaire response rates were acceptable.

The response rate of the knowledge test at the end of the semester was low (53%) as filling out the test was voluntary. Moreover, only 34% of students taking part in VDTs completed the knowledge test compared to 67% of students participating in the conventional BTs. In addition, the multiple-choice test measures theoretical knowledge and does not give enough information about practical skills and attitudes acquired during the practices. We also need to highlight that more time elapsed between the traditional BTs and the knowledge test than between VDTs and the knowledge test. The knowledge test was accessible when all students completed the thematic practices to avoid wide distribution of the test questions within the student body.

Despite these limitations our investigation gives new information about the implementation of VDT to keep undergraduate intensive therapy and anaesthesiology education continuous during a pandemic.

## Conclusion

Our cross-sectional study demonstrates that virtual distance education is effective in maintaining education of intensive therapy and anaesthesiology continuous among fifth-year medical students during the COVID-19 outbreak. Students and instructors participating in our rapidly introduced VDTs during the restrictions due to COVID-19 outbreak found the new method acceptable. However, BTs, personal communication, interactivity and experiencing ICU atmosphere play a crucial role in teaching intensive therapy and anaesthesiology and should still be included in the undergraduate educational practice.

## Supplementary Information

**Additional file 1 **STROBE Statement—Checklist of items that should be included in reports of ***cross-sectional studies.***

**Additional file 2: Table 1.** Practical curriculum of traditional undergraduate education in intensive therapy and anaesthesiology at Semmelweis University. **Table 2.** Students' questionnaire. **Table 3.** Instructors' questionnaire. **Table 4.** Steps of questionnaire development.

**Additional file 3: Table 1.** Description of binary logistic regression analysis performed to determine if students' previous job experience with critically ill patients has an influence on their self-reported preparedness. **Fig. 1.** The relationship between previous job experience with critically ill patients regularly and self-preparedness in recoginzing patients with respiratory failure, shock or being aware of perioperative management.

## Data Availability

The datasets generated and analyzed during the current study are available in the Synapse repository, https://www.synapse.org/#!Synapse:syn22496218/files/.

## Abbreviations

BT: Bed-side training
COVID-19: Coronavirus disease 2019
ICU: Intensive care unit
ITA: Intensive therapy and anaesthesiology
IQR: Interquartile range
VDT: Virtual distance training
