# Supplementary material for: The efficacy of virtual distance training of intensive therapy and anaesthesiology among fifth-year medical students during the COVID-19 pandemic: a cross-sectional study
Source: BMC Med Educ. 2021 Jul 22;21:393. doi: 10.1186/s12909-021-02826-1 (PMC8295977; doi:10.1186/s12909-021-02826-1)
Supplement: Supplementary file 2 — Additional file 2: Table 1. Practical curriculum of traditional undergraduate education in intensive therapy and anaesthesiology at Semmelweis University. Table 2. Students' questionnaire. Table 3. Instructors' questionnaire. Table 4. Steps of questionnaire development. [file 12909_2021_2826_MOESM2_ESM.pdf]

Additional file 2 - **Table 1.** Practical curriculum of traditional undergraduate education in intensive therapy and anaesthesiology at Semmelweis University.

**Practice name**	**Topics covered during practice**
Introduction	Students visited the intensive care unit (ICU). The principles of patient admission to the unit, examination of patients in the ICU and equipment used during patient management were introduced.
Respiratory practice	Recognizing respiratory insufficiency, therapeutic options of respiratory diseases requiring intensive therapy and the principles of mechanical ventilation were presented to students. The practices were held in the ICU.
Shock practice	Students observed patients in the ICU and focused on learning the recognition of shock, the principles of hemodynamic monitoring and hemodynamic management.
Anaesthesia practice	Students visited an operating room without an ongoing operation. They were introduced to the principles of perioperative management, monitoring during anaesthesia and anaesthesia techniques. They could observe an anaesthesia machine.
Trauma practice	The students observed trauma patients in the ICU.
Simulation sessions	The principles of basic life support, advanced life support and the management of critically ill patients were practiced. The tools of low and high-fidelity simulation were also used during the simulation trainings.

Additional file 2 - **Table 2.** Students' questionnaire.

**Likert-scale questions**	Question	Possible answers
1	I received from the virtual distance trainings exactly what I have expected.	1: strongly disagree; 2: disagree; 3: undecided; 4: agree; 5: strongly agree
2	The virtual practices perfectly replaced the bed-side sessions – there is no need for the latter.	1: strongly disagree; 2: disagree; 3: undecided; 4: agree; 5: strongly agree
3	The virtual practices are useful but strictly in combination with bed-side sessions.	1: strongly disagree; 2: disagree; 3: undecided; 4: agree; 5: strongly agree
4	I can recognize a patient with respiratory insufficiency after completing the virtual training.	1: strongly disagree; 2: disagree; 3: undecided; 4: agree; 5: strongly agree
5	I can recognize a patient with shock after completing the virtual training.	1: strongly disagree; 2: disagree; 3: undecided; 4: agree; 5: strongly agree
6	I am aware of the perioperative patient management after completing the virtual training.	1: strongly disagree; 2: disagree; 3: undecided; 4: agree; 5: strongly agree
7	I was disadvantaged by taking the virtual practices instead of the bed-side sessions.	1: strongly disagree; 2: disagree; 3: undecided; 4: agree; 5: strongly agree
8	To what extent have the virtual practices raised your interest in intensive therapy and anaesthesiology?	1: definitely not; 2: probably not; 3: undecided; 4: probably yes; 5: definitely yes
9	To what extent was it easy to follow the flow of the virtual practices?	1: definitely not; 2: probably not; 3: undecided; 4: probably yes; 5: definitely yes
10	The virtual training was overall effective.	1: strongly disagree; 2: disagree; 3: undecided; 4: agree; 5: strongly agree
11	To what extent have you found the virtual practices' imaging acceptable?	1: definitely not; 2: probably not; 3: undecided; 4: probably yes; 5: definitely yes
12	To what extent have you found the virtual practices' audio acceptable?	1: definitely not; 2: probably not; 3: undecided; 4: probably yes; 5: definitely yes
13	To what extent have you found the material on Moodle® platform helpful?	1: definitely not; 2: probably not; 3: undecided; 4: probably yes; 5: definitely yes

14	To what extent have you found the virtual trainings acceptable in total?	1: definitely not; 2: probably not; 3: undecided;<br>4: probably yes; 5: definitely yes
**Open-ended questions**	Question	
1	What are the three main things you have learned during our virtual distance trainings?	
2	What did you like in your favourite virtual training during our virtual education?	
**Multiple choice questions**	Question	Possible answers
1	Age	
2	Gender	
3	Have you worked in the health care during your studies?	a. Regularly - at least once in a week b. Rarely - less than once a week c. No
4	Have you worked at an ICU, ED or ambulance service during your studies?	a. Regularly - at least once in a week b. Rarely - less than once a week c. No

Additional file 2 - **Table 3**. Instructors' questionnaire.

**Likert-scale questions**	Question	Possible answers
1	I received sufficient information regarding distance learning from my institution.	1: strongly disagree; 2: disagree; 3: undecided; 4: agree; 5: strongly agree
2	I received sufficient materials regarding the virtual course from my institution.	1: strongly disagree; 2: disagree; 3: undecided; 4: agree; 5: strongly agree
3	Plenty of technical problems arose during virtual education leading to quality decrease of our teaching.	1: strongly disagree; 2: disagree; 3: undecided; 4: agree; 5: strongly agree
4	I received sufficient technical support during virtual education from my institution.	1: strongly disagree; 2: disagree; 3: undecided; 4: agree; 5: strongly agree
5	I could achieve a good interaction with students during virtual education – the same as during in-person sessions.	1: strongly disagree; 2: disagree; 3: undecided; 4: agree; 5: strongly agree
6	Students` interactivity was satisfactory during virtual sessions – it reached the level of an in-person session.	1: strongly disagree;

		2: disagree; 3: undecided; 4: agree; 5: strongly agree
7	I could transfer the planned knowledge during virtual teaching.	1: strongly disagree; 2: disagree; 3: undecided; 4: agree; 5: strongly agree
8	The students were disadvantaged by taking the virtual practices instead of the bed-side sessions.	1: strongly disagree; 2: disagree; 3: undecided; 4: agree; 5: strongly agree
9	I enjoyed virtual distance training despite the difficulties of the new situation caused by the COVID-19 outbreak.	1: strongly disagree; 2: disagree; 3: undecided; 4: agree; 5: strongly agree
10	The virtual practices perfectly replaced the bed-side sessions – there is no need for the latter.	1: strongly disagree; 2: disagree; 3: undecided; 4: agree; 5: strongly agree
11	The virtual practices are useful but strictly in combination with the bed-side sessions.	1: strongly disagree; 2: disagree; 3: undecided; 4: agree; 5: strongly agree

12	The virtual training was overall effective.	1: strongly disagree; 2: disagree; 3: undecided; 4: agree; 5: strongly agree
13	The virtual sessions should be built into our curriculum in the future.	1: strongly disagree; 2: disagree; 3: undecided; 4: agree; 5: strongly agree
14	I would never teach at virtual distance learning again.	1: strongly disagree; 2: disagree; 3: undecided; 4: agree; 5: strongly agree
**Open-ended questions**	Question	
1	List at least two advantages of virtual distance learning	
2	List at least two disadvantages of virtual distance learning	
**Multiple choice questions**	Question	Possible answers
1	Age	
2	Gender	
3	Job description	a. Resident<br>b. Specialist<br>c. Academic position<br>d. Other
4	Language program	a. Hungarian<br>b. German<br>c. Both
5	How long have you been involved in the education of medical students?	a. 0-5 years<br>b. >5 years<br>c. >10 years

6	Do you use e-learning as an educator?	a. Yes, regularly - at least once in a week<br>b. Yes, rarely - less than once a week<br>c. No

Additional file 2 - **Table 4.** Steps of questionnaire development.

	**Students' questionnaire**	**Instructors' questionnaire**
**Step 1**	Development of questionnaire	
**Step 2**	Review by 3 senior educators of our institute – if items were clear and written in accordance with current best practices in questionnaire design.	
**Step 3**	6 educators taking part in our virtual distance training assessed content, relevance and intelligibility of items.	6 educators outside from our institute experienced in virtual distance education assessed content, relevance and intelligibility of items.
**Step 4**	10 Hungarian and 5 German students went through a written cognitive interview to ensure responders' proper interpretation followed by a pilot.	6 instructors of our institute went through a written cognitive interview to ensure responders' proper interpretation followed by a pilot.
**Step 5**	Students participating in virtual distance trainings received the questionnaire link via e-mail at the end of their virtual practice block.	Instructors participating in virtual distance trainings received the questionnaire link via e-mail at the end of the semester.
**Step 6**	Students received a reminder three times via e-mail: at the end of the semester, at the middle of the exam period and two days before their oral exam.	Instructors received a reminder one week later via e-mail and two weeks later via phone call.
