# Supplementary material for: The efficacy of virtual distance training of intensive therapy and anaesthesiology among fifth-year medical students during the COVID-19 pandemic: a cross-sectional study
Source: BMC Med Educ. 2021 Jul 22;21:393. doi: 10.1186/s12909-021-02826-1 (PMC8295977; doi:10.1186/s12909-021-02826-1)
Supplement: Supplementary file 3 — Additional file 3: Table 1. Description of binary logistic regression analysis performed to determine if students' previous job experience with critically ill patients has an influence on their self-reported preparedness. Fig. 1. The relationship between previous job experience with critically ill patients regularly and self-preparedness in recoginzing patients with respiratory failure, shock or being aware of perioperative management. [file 12909_2021_2826_MOESM3_ESM.pdf]

Additional file 3 - **Table 1.** Description of binary logistic regression analysis performed to determine if students' previous job experience with critically ill patients has an influence on their self-reported preparedness

Variable	Question	Answer
**Dependent variables**	I can recognize a patient with respiratory insufficiency after completing the virtual course.<br><br>I can recognize a patient with shock after completing the virtual course.<br><br>I am aware of perioperative patient management after completing the virtual course.	1: strongly disagree;<br>2: disagree;<br>3: undecided;<br>4: agree;<br>5: stronlgy agree
**Independent variable**	Previous job experience with critically ill patients	a. Regularly - at least once in a week b. Rarely - less than once a week c. No

Notes: Dummy categories were created from the Likert-scale answers to allow binary logistic regression analysis. Answers 1,2 and 3 were grouped to category 0 representing disagreement, and answers 4 and 5 (where the responders showed clear confidence) were categorized as 1 representing the agreement with the above mentioned statements.

Additional file 3 - **Fig. 1.** The relationship between previous job experience with critically ill patients regularly and self-preparedness in recoginzing patients with respiratory failure, shock or being aware of perioperative management.

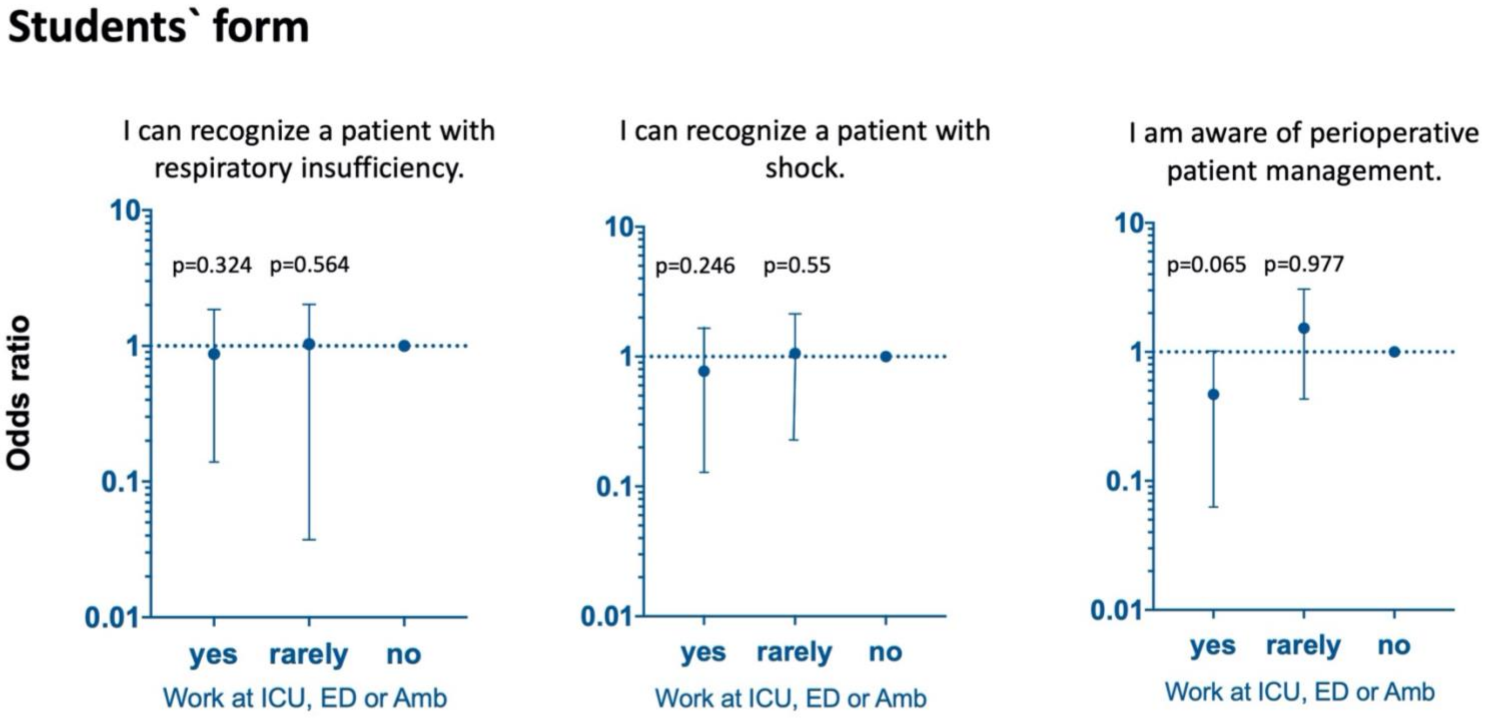

Binary logistic regression was performed. CI: confidence interval; ICU: intensive care unit; EMS: emergency department; EMS: Emergency Medical System. Answer "no" represents reference category.
